# ^177^Lu Anti-Angiogenic Radioimmunotherapy Targeting ATP Synthase in Gastric Cancer Model

**DOI:** 10.3390/antib13030051

**Published:** 2024-06-27

**Authors:** Bok-Nam Park, Young-Sil An, Su-Min Kim, Su-Jin Lee, Yong-Jin Park, Joon-Kee Yoon

**Affiliations:** Department of Nuclear Medicine & Molecular Imaging, Ajou University School of Medicine, Worldcup-ro 164, Suwon 16499, Republic of Korea; curies@ajou.ac.kr (B.-N.P.); aysays@aumc.ac.kr (Y.-S.A.); smversion@naver.com (S.-M.K.); suesj202@aumc.ac.kr (S.-J.L.); yjpark@aumc.ac.kr (Y.-J.P.)

**Keywords:** radioimmunotherapy, ATP synthase, ^177^Lu, angiogenesis, gastric cancer

## Abstract

This study investigated a novel radioimmunotherapy strategy for targeting tumor angiogenesis. We developed a radiopharmaceutical complex by labeling an anti-adenosine triphosphate synthase (ATPS) monoclonal antibody (mAb) with the radioisotope ^177^Lu using DOTA as a chelating agent. ^177^Lu-DOTA-ATPS mAb demonstrated high labeling efficiency (99.0%) and stability in serum. MKN-45 cancer cells exhibited the highest cellular uptake, which could be specifically blocked by unlabeled ATPS mAb. In mice, ^177^Lu-DOTA-ATPS mAb accumulated significantly in tumors, with a tumor uptake of 16.0 ± 1.5%ID/g on day 7. ^177^Lu-DOTA-ATPS mAb treatment significantly reduced the viability of MKN-45 cells in a dose-dependent manner. In a xenograft tumor model, this radioimmunotherapy strategy led to substantial tumor growth inhibition (82.8%). Furthermore, combining ^177^Lu-DOTA-ATPS mAb with sunitinib, an anti-angiogenic drug, enhanced the therapeutic efficacy of sunitinib in the mouse model. Our study successfully developed ^177^Lu-DOTA-ATPS mAb, a radioimmunotherapy agent targeting tumor blood vessels. This approach demonstrates significant promise for inhibiting tumor growth, both as a single therapy and in combination with other anti-cancer drugs.

## 1. Introduction

Angiogenesis, the formation of new blood vessels, is a crucial process for normal tissue growth and tumor expansion. Disrupting this process has become a major focus for researchers aiming to develop effective cancer therapies. Tumors hijack angiogenesis to fuel their growth and metastasis [1]. This uncontrolled vessel formation is countered by endogenous inhibitors like angiostatin. Angiostatin maintains a balanced angiogenic environment by suppressing the effects of proangiogenic factors like vascular endothelial growth factor and fibroblast growth factor [2]. While typically residing within the mitochondrial inner membrane, adenosine triphosphate synthase (ATPS), an enzyme responsible for ATP generation, has been found on the surface of endothelial and tumor cells, called “ectopic” ATPS [3]. Specific subunits (α/β) of this ectopic ATPS serve as binding sites for angiostatin [4]. Antibodies developed against specific ATPS subunits can directly inhibit the enzyme’s activity on the endothelial cell surface, which, in turn, disrupts the formation of new blood vessels and directly hinders the proliferation and migration of cancer cells [5,6,7]. We also have shown that radiolabeled anti-ATPS antibodies are promising tools for radioimmunotherapy and immuno-positron emission tomography (PET) imaging. Radioiodine (^131^I)-labeled anti-ATPS antibody effectively suppressed the tumor growth by 2.5-fold in a gastric cancer model [8]. Anti-ATPS antibodies labeled with Zirconium-89 (^89^Zr) demonstrated significantly higher targeting specificity for MDA-MB-231 tumors with abundant ectopic ATPS expression compared to PC-3 tumors with low expression [9]. Given its role in tumor angiogenesis, ectopic ATPS emerges as a novel target for developing effective anti-angiogenic therapies.

Since the landmark report in 1981 on a successful radiolabeled antibody targeting carcinoembryonic antigen [10], radioimmunotherapy has remained a beacon of hope in the fight against cancer, offering a powerful tool for targeted therapy. Zevalin™ and Bexxar™, being used for the treatment of non-Hodgkin’s lymphoma, are prime examples of radioimmunotherapy successfully employed in modern medicine. Lutetium-177 (^177^Lu) has gained widespread popularity as a preferred radioisotope in recent years. This radioisotope demonstrates its versatility in cancer treatment. Studies have shown its effectiveness in treating two distinct cancers: unresectable metastatic neuroendocrine tumors [11] and metastatic castration-resistant prostate cancer [12]. In both cases, it is used as a targeted therapy approach (peptide receptor radionuclide therapy and radioligand therapy). ^177^Lu has favorable characteristics for radioimmunotherapy. It emits both high-energy β-ray (Emax = 761 KeV) and γ-rays (113 in 6.4% and 208 KeV in 13%) and decays with a half-life of 6.7 days [13], which is favorable for radioimmunotherapy.

This study aims to develop a new radioimmunotherapy approach that specifically targets tumor angiogenesis. We synthesized ^177^Lu-labeled anti-ATPS antibody to achieve this and evaluated its antitumor efficacy in a gastric cancer animal model.

## 2. Materials and Methods

### 2.1. Radiosynthesis of ^177^Lu-DOTA-ATPS mAb

**Figure 1:**
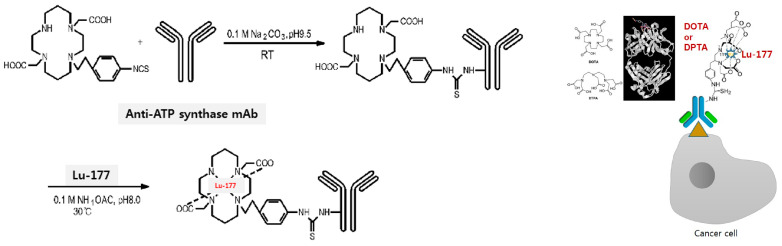
Schematic diagram for radiosynthesis of ^177^Lu-DOTA-ATPS mAb.

The ATPS mAb was purchased from Abcam (ab14730, MW 52 kDa; Cambridge, MA, USA) and stored as aliquots at −78 °C. ^177^LuCl_3_ was obtained from PLATOM (National Centre for Nuclear Research, Poland). For conjugation, a 50-fold molar excess of p-SCN-Bn-tetraazacyclododecane-1,4,7,10-tetraacetic acid (DOTA, Futurechem, Seoul, Republic of Korea) in 30 µL dimethyl sulfoxide was added to the ATP mAb (100–200 µg in 20 µL 0.1 N NaHCO_3_ buffer), and the reaction mixture was incubated at 37 °C for 30 min. After incubation, the conjugation mixture was purified by using Slide-A-Lyzer™ Dialysis Cassettes (2K MWCO, Thermo Fisher Scientific, Rockford, IL, USA) to remove unconjugated *p*-SCN-Bn-DOTA. ^177^LuCl_3_ (37–111 MBq/10 μL) was buffered with 0.5 M NH_4_OAc (pH 5.5), followed by 100–200 µg DOTA-mAb. The reaction mixture was incubated at 37 °C for 1 h [14]. After completion of synthesis, the reaction mixture was purified on a size-exclusive PD-10 column (GE Healthcare) and ^177^Lu-DOTA-ATPS mAb was separated by eluting with PBS (Figure 1). For analysis of labeled ^177^Lu-DOTA-ATPS mAb, it was spotted on silica gel impregnated aluminum sheets (Merck, Darmstadt, Germany), developed with 0.02 M citrate buffer, and confirmed by using radio-thin-layer chromatography (radio-TLC) (Bioscan, Eckert & Ziegler Radiopharma Inc., Wilmington, MA, USA). In vitro stability was measured in triplicates at 2 h, 24 h, and on the 7th day, in PBS or serum, at 4 °C, room temperature, or 37 °C.

### 2.2. Cancer Cell Culture and Xenograft Tumor Model

All cancer cell lines, human breast adenocarcinoma (MDA-MB-231), human fibrosarcoma (HT-1080), human lung adenocarcinoma (A549), human follicular thyroid carcinoma (FTC-133), human prostate adenocarcinoma (PC-3), and human gastric adenocarcinoma (MKN-45) were purchased from the Korean Cell Line Bank (Seoul, Republic of Korea). 

All cells except FTC-133 (FTC-133 cells were cultured in DMEM/F-12, 1:1 mixture, 10% fetal bovine serum, WelGENE Inc., Daegu, Republic of Korea) were cultured with RPMI-1640 medium (WelGENE) supplemented with 10% FBS and 1% penicillin/streptomycin (WelGENE) at 37 °C and 5% fully humidified CO_2_. Animal experiments were performed according to protocols approved by the Care of Experimental Animals Committee (IACUC No. 2021-0067). Six-week-old female Balb/c nude mice (Orient Bio, Seongnam, Republic of Korea) were maintained under specific pathogen-free conditions [15]. To create a tumor xenograft model, 5 × 10^6^ tumor cells were mixed with phenol red-free Matrigel (Corning^®^, Bedford, MA, USA) and subcutaneous inoculation was injected into the right shoulder of each mouse. Experiments were performed about 10 to 14 days after injection of cells, when tumors reached a diameter of approximately 5 to 10 mm. 

### 2.3. Cellular Uptake of ^177^Lu-DOTA-ATPS mAb in Various Cancer Cells 

Cellular uptake of ^177^Lu-DOTA-ATPS mAb was measured in the six human cancer cell lines as described previously [8]. In brief, 5 × 10^5^ cells were seeded per well in 12-well plates and cultured for 24 h. Upon attachment, 37 kBq ^177^Lu-DOTA-ATPS mAb was added to freshly replaced culture media, followed by incubation of the cells for 1, 4, or 24 h at 37 °C and 5% CO_2_. After incubation, the cells were washed twice with cold PBS and harvested with 0.1 N NaOH. Radioactivity of the cells was counted using a Gamma-HEs gamma counter (Shinjin Medics Inc., Goyang, Republic of Korea) and normalized to the cell protein content obtained using the Bradford method [16]. Data are presented as the percentage of 1 h uptake.

### 2.4. Specific Binding of ^177^Lu-DOTA-ATPS mAb in MKN-45 Cells 

MKN-45 cells were cultured in 12-well plates and incubated with 111 kBq free ^177^Lu, ^177^Lu-DOTA-ATPS mAb, or ^177^Lu-DOTA-IgG for 24 h at 37 °C and 5% CO_2_. After incubation, the cells were washed twice with cold PBS, harvested with 0.1 N NaOH, and the radioactivity was counted using a gamma counter. The cell protein content was determined using the Pierce 660^TM^ Protein Assay Kit for normalization (Thermo Fisher Scientific, Rockford, IL, USA). Cellular uptake was expressed as a percentage of free ^177^Lu uptake. 

### 2.5. Competitive Binding of ^177^Lu-DOTA-ATPS mAb in MKN-45 Cells 

Competitive inhibition of ^177^Lu-DOTA-ATPS mAb binding was examined using unlabeled ATPS mAb in MKN-45 cells. Cells were cultured in 12-well plates and treated with 10% FBS and 1% penicillin/streptomycin at 37 °C and 5% CO_2_ (as described previously). The cells were pretreated with 6.4 μM unlabeled ATPS mAb for 1 h, while control cells were incubated with the vehicle. Then, 111 kBq ^177^Lu-DOTA-ATPS mAb was added to the cells and incubated for 4 or 24 h under the same conditions. After incubation, cellular uptake was calculated and expressed as a percentage relative to that of untreated control. Cellular uptake, specific binding, and inhibition experiments were all performed in triplicate.

### 2.6. ^177^Lu-DOTA-ATPS mAb Radioimmunotherapy in MKN-45 Cells 

MKN-45 cells were cultured in 96-well plates and treated with 3.7 or 7.4 MBq ^177^Lu-DOTA-ATPS mAb, unlabeled ATPS mAb, or left untreated for 24 h at 37 °C and 5% CO_2_. To investigate the effect of combination therapy, MKN-45 cells were treated with either 7.4 MBq ^177^Lu-DOTA-ATPS mAb or 5 mg/mL sunitinib (LC Laboratories^®®^, Woburn, MA, USA), both, or left untreated for 24 h at 37 °C and 5% CO_2_ [17]. After incubation, cell viability was measured using an XTT assay kit (Cayman Chemical, Ann Arbor, MI, USA) with a further 2 hr incubation at 37 °C. Absorbance was read at 450 nm using a microplate reader (Bio-Rad Laboratories Inc., Hercules, CA, USA). 

### 2.7. Biodistribution Study of Wild-Type Mice and MKN-45 Tumor Xenograft Models 

Wild-type mice and MKN-45 tumor-bearing mice (n = 4–5 per time point) were intravenously injected with 3.7 MBq ^177^Lu-DOTA-ATPS mAb, ^177^Lu-IgG, or free ^177^Lu. The mice were then anesthetized, sacrificed, and dissected for organ radioactivity analysis at 1, 2, 4, and 7 days after injection. Major organs (heart, lung, liver, spleen, stomach, kidneys, intestine, muscle, and bone), blood, and tumors were dissected, weighed, and counted for radioactivity using a gamma counter. Uptake in the organs and tumors was expressed as the percentage of the injected dose per gram of tissue (%ID/g).

### 2.8. Blocking Study of ^177^Lu-DOTA-ATPS mAb in MKN-45 Tumor-Bearing Mice 

To investigate blocking efficacy, 50 µg of unlabeled ATPS mAb was co-injected with 3.7 MBq ^177^Lu-DOTA-ATPS mAb (1 µg as mAb) through the tail vein (n = 2). Tumors and organs were then removed at 1 and 7 days after injection for subsequent radioactivity analysis. The organs were weighed and counted for radioactivity using a gamma counter. Results were expressed as %ID/g. 

### 2.9. Radioimmunotherapy, Immunohistochemical Staining, and ^18^F-FDG-PET Imaging in MKN-45 Tumor-Bearing Mice

To investigate radioimmunotherapy, tumor models were intravenously injected with 18.5 MBq ^177^Lu-DOPA-ATPS mAb, 30 μg unlabeled ATPS mAb (10 times larger than the therapeutic dosage of ^177^Lu-DOPA-ATPS mAb), 30 μg unlabeled IgG, and vehicle (normal saline), respectively, once a week for 4 weeks (n = 4 for each group) [8]. Tumor size was measured twice a week in two dimensions (length and width). Tumor volume was calculated using the formula, V = (length ×. width^2^)/2, and compared among the groups [18]. Tumor growth inhibition (TGI, %) was calculated using the formula, TGI = (1 − mean volume of treated tumors/mean volume of control tumors) × 100.

The effect of combination therapy of ^177^Lu-DOTA-ATPS mAb and sunitinib was evaluated in mice bearing MKN-45 tumors. Mice were divided into four groups: vehicle (0.9% NaCl), 18.5 MBq ^177^Lu-DOTA-ATPS mAb alone, 40 mg/kg sunitinib alone, or a combination of both (n = 6 for each group). Mice received 18.5 MBq ^177^Lu-DOTA-ATPS mAb and 40 mg/kg sunitinib once a week for 4 weeks [19]. Tumor size was measured twice a week as described above. Body weights were measured once a week. 

For imaging, PET images were acquired 1 h after intravenous injection of 18.5 MBq 2-deoxy-2-[^18^F]fluoro-D-glucose (^18^F-FDG) using a PET scanner (SimPET, BRIGHTONIX IMAGING^®^, Seoul, Republic of Korea) before and after therapies. Mice were scanned for 20 min under anesthesia with isoflurane inhalation. 

For immunohistochemistry, tumors were dissected immediately after the PET imaging at the 4th week. Slides were stained using an anti-CD31 antibody (ab28364, abcam) according to the manufacturer’s standard procedure.

### 2.10. Statistical Analysis

All data are presented as means ± standard errors. The statistical comparison of cellular uptake and tumor size was evaluated by Student’s *t*-test and Kruskal–Wallis test using statistical software (R, version 3.1.2), and the difference was considered significant at *p* < 0.05.

## 3. Results

### 3.1. Labeling Efficiency and In Vitro Stability of ^177^Lu-DOTA-ATPS mAb

**Figure 2:**
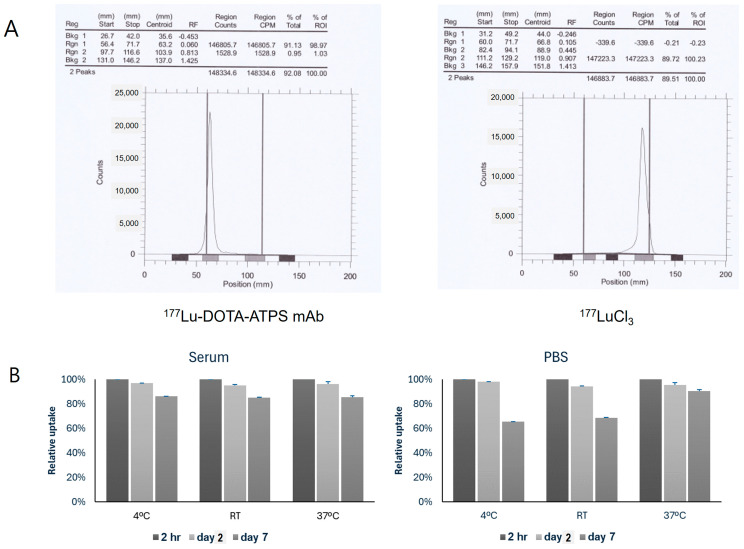
Labeling efficiency (**A**) and in vitro stability (**B**) of ^177^Lu-DOTA-ATPS mAb. The Rf value of ^177^Lu-DOTA-ATPS mAb was between 0.01 and 0.05, while that of ^177^LuCl_3_ was between 0.6 and 1.0. The in vitro stabilities of ^177^Lu-DOTA-ATPS mAb in serum remained unchanged up to 7 days. DOTA, tetraazacyclododecane-1,4,7,10-tetraacetic acid; ATPS, adenosine triphosphate synthase; mAb, monoclonal antibody; RT, room temperature; PBS, phosphate-buffered saline.

The ^177^Lu-DOTA-ATPS mAb was successfully synthesized according to the schematic representation. The radiochemical yield of ^177^Lu-DOTA-ATPS mAb was 99.0% (Figure 2A). The in vitro stabilities of ^177^Lu-DOTA-ATPS mAb in serum were at least 95% on the 2nd day and 85% on the 7th day at all temperature conditions (Figure 2B). Similarly, the in-vitro stabilities of ^177^Lu-DOTA-ATPS mAb in PBS were at least 94% on the 2nd day, regardless of temperature. However, on the 7th day, the stability dropped significantly to 65%, 68%, and 91% at 4 °C, room temperature, and 37 °C, respectively (all *p* < 0.005).

### 3.2. Cellular Uptake, Specific Binding, and Inhibition of ^177^Lu-DOTA-ATPS mAb

**Figure 3:**
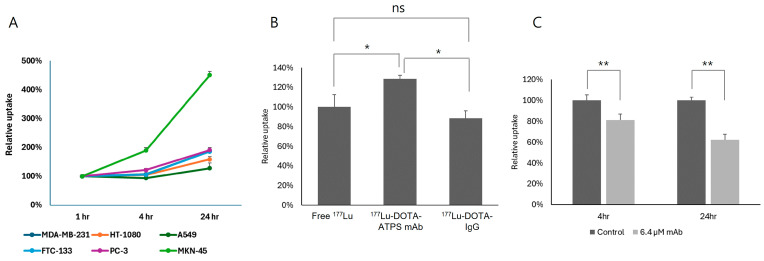
The cellular uptake (**A**), specific binding (**B**), and inhibition study (**C**) of ^177^Lu-DOTA-ATPS mAb. MKN-45 cells showed the highest cellular uptake of ^177^Lu-DOTA-ATPS mAb among the tested cancer cell lines. ^177^Lu-DOTA-ATPS mAb uptake was specific and inhibited by unlabeled ATPS mAb in MKN-45 cells. DOTA, tetraazacyclododecane-1,4,7,10-tetraacetic acid; ATPS, adenosine triphosphate synthase; mAb, monoclonal antibody. *p* < 0.05 *, *p* < 0.005 **, ns: not significant.

Six cancer cell lines were evaluated for their cellular uptake of ^177^Lu-DOTA-ATPS (Figure 3A). MKN-45 cells exhibited a time-dependent increase in uptake reaching 189.3% ± 9.8% and 450.8% ± 13.3% of the 1 hr uptake at 4 and 24 h, respectively (*p* < 0.0005 and *<* 0.0001, respectively). PC-3 also showed increased uptake at both 4 h (121.9% ± 4.9%, *p* < 0.05) and at 24 h (190.7% ± 8.8%, *p* < 0.0005). Similarly, MDA-MB-231 (186.4% ± 7.7%, *p* < 0.001), HT-1080 (158.7% ± 9.5%, *p* < 0.005), A549 (128.0% ± 9.9%, *p* < 0.05), and FTC-133 (186.2% ± 4.9%, *p* < 0.001) showed a significant increase in cellular uptake of ^177^Lu-DOTA-ATPS mAb at 24 h, while their 4 hr uptake remained unchanged (*p* > 0.05). Notably, MKN-45 cells demonstrated significantly higher uptake compared to other cell lines at 24 h (all *p* < 0.0005). This finding prompted further cellular and animal studies using MKN-45 cells.

In MKN-45 cells, the uptake of ^177^Lu-DOTA-ATPS mAb was compared with that of free ^177^Lu (^177^LuCl_3_) and ^177^Lu-DOTA-IgG at 24 h (Figure 3B). The cellular uptake of ^177^Lu-DOTA-ATPS mAb (128.7% ± 3.6%) was significantly higher than that of free ^177^Lu and ^177^Lu-DOTA-IgG (88.5% ± 7.8%) (all *p* < 0.05). There was no significant difference in cellular uptake between free ^177^Lu and ^177^Lu-DOTA-IgG (*p* > 0.05).

The uptake of ^177^Lu-DOTA-ATPS mAb was inhibited by a high dose of unlabeled ATPS mAb at both 4 h (81.0% ± 5.9%, *p* < 0.005) and at 24 h (62.2% ± 5.4%, *p* < 0.005) (Figure 3C). The inhibitory effect of unlabeled ATPS mAb was more pronounced at 24 h compared to 4 h (*p* < 0.05).

### 3.3. ^177^Lu-DOTA-ATPS mAb Radioimmunotherapy in MKN-45 Cells

**Figure 4:**
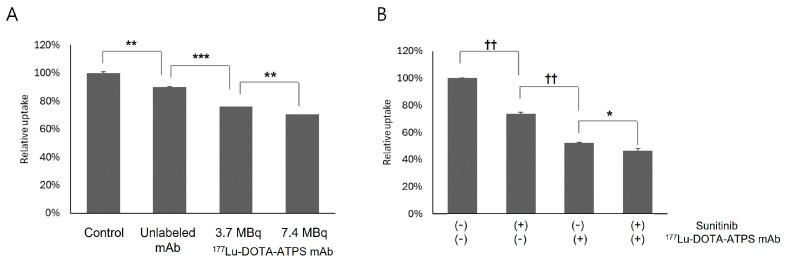
Radioimmunotherapy with ^177^Lu-DOTA-ATPS mAb alone (**A**) and ^177^Lu-DOTA-ATPS mAb in combination with sunitinib (**B**) in MKN-45 cells. DOTA, tetraazacyclododecane-1,4,7,10-tetraacetic acid; ATPS, adenosine triphosphate synthase; mAb, monoclonal antibody. *p* < 0.05 *, *p* < 0.005 **, *p* < 0.001 ***, *p* < 0.0005 ^+^, *p* < 0.00005 ^++^.

Radioimmunotherapy with ^177^Lu-DOTA-ATPS mAb significantly reduced cell viability (Figure 4A) compared with vehicle-treated controls. Treatment with 3.7 MBq (78.9% *±* 1.2%, *p* < 0.005) and 7.4 MBq (70.4% ± 1.7%, *p* < 0.01) of ^177^Lu-DOTA-ATPS mAb resulted in a dose-dependent decrease in viable cells (*p* < 0.005). Unlabeled ATPS mAb also significantly reduced cell viability (89.8% ± 0.5%, *p* < 0.001 vs. control); however, the therapeutic effect of either dose of ^177^Lu-DOTA-ATPS mAb was significantly greater (all *p* < 0.0001).

An anti-angiogenic therapy with sunitinib (5 mg/mL) significantly decreased the number of viable MKN-45 cells (73.8% ± 1.0% of controls, *p* < 0.00005) (Figure 4B). Combination therapy with 7.4 MBq of ^177^Lu-DOTA-ATPS mAb and sunitinib showed a greater reduction in cell viability (46.5% ± 1.5%) compared to single therapy with ^177^Lu-DOTA-ATPS mAb (52.5% ± 0.2%, *p* < 0.05) or sunitinib (*p* < 0.00005). Notably, among single therapies, 7.4 MBq of ^177^Lu-DOTA-ATPS mAb exhibited a greater therapeutic effect than 5 mg/mL sunitinib (*p* < 0.00005).

### 3.4. Biodistribution of ^177^Lu-DOTA-ATPS mAb in Wild-Type Mice and MKN-45 Tumor Xenograft Models

**Figure 5:**
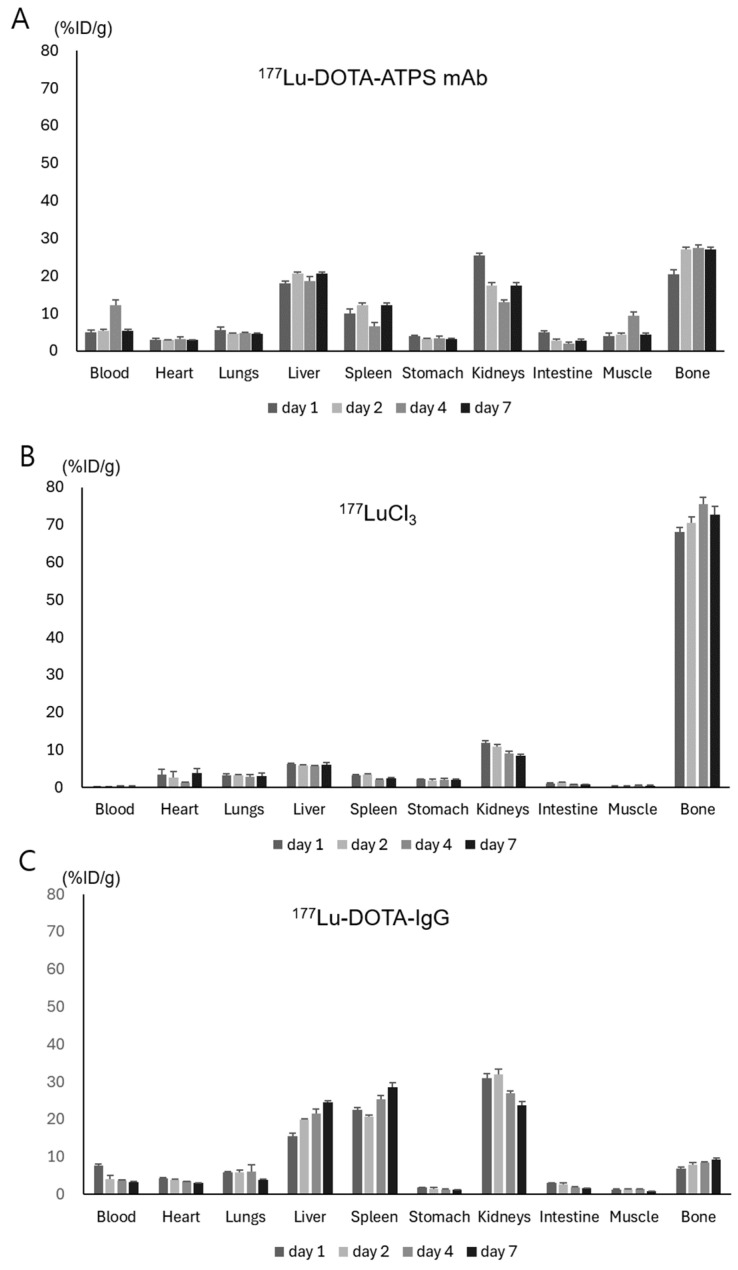
Biodistribution of ^177^Lu-DOTA-ATPS mAb (**A**), ^177^LuCl_3_ (**B**), and ^177^Lu-DOTA-IgG (**C**) in wild-type mice on days 1, 2, 4, and 7. DOTA, tetraazacyclododecane-1,4,7,10-tetraacetic acid; ATPS, adenosine triphosphate synthase; mAb, monoclonal antibody.

**Figure 6:**
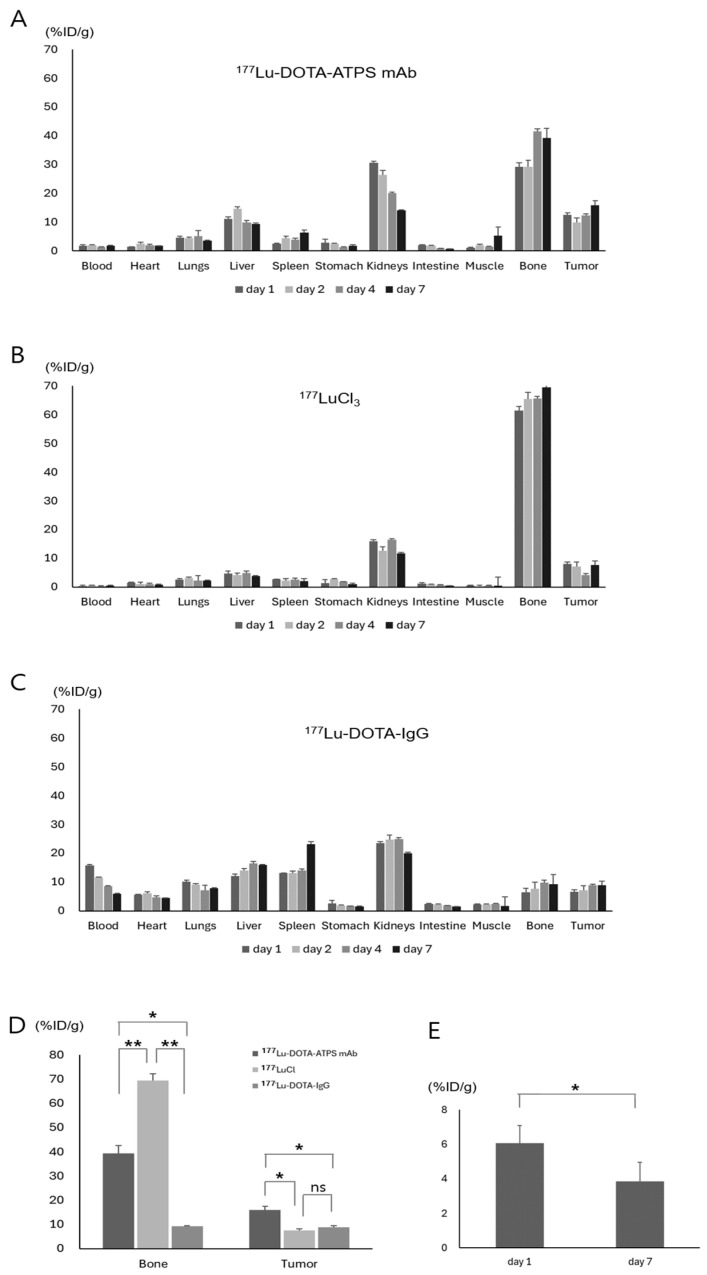
Biodistribution of ^177^Lu-DOTA-ATPS mAb (**A**), ^177^LuCl_3_ (**B**), and ^177^Lu-DOTA-IgG (**C**) in mice bearing MKN-45 tumors on day 1, 2, 4, and 7. Comparison of bone marrow and tumor uptake among radiopharmaceuticals (**D**). Inhibition of ^177^Lu-DOTA-ATPS mAb uptake in tumors by unlabeled ATPS mAb (**E**). DOTA, tetraazacyclododecane-1,4,7,10-tetraacetic acid; ATPS, adenosine triphosphate synthase; mAb, monoclonal antibody. *p* < 0.05 *, *p* < 0.005 **, ns: not significant.

The biodistribution of ^177^Lu-DOTA-ATPS mAb was evaluated in wild-type mice (Figure 5) and mice bearing MKN-45 tumors (Figure 6) on days 1, 2, 4, and 7. In wild-type mice, bone marrow uptake of ^177^Lu-DOTA-ATPS mAb reached 20.4 ± 1.3%ID/g on day 1 and remained stable from day 2 to day 7. Renal uptake was highest on day 1 (25.4 ± 0.6%ID/g) and decreased slightly from day 2 to day 7 (13.0 to 17.4%ID/g). Hepatic uptake ranged from 17.9 to 20.6%ID/g.

In contrast, ^177^LuCl_3_ primarily accumulated in the bone marrow (68.1 ± 1.2%ID/g, 70.4 ± 1.6%ID/g, 75.6 ± 1.7%ID/g, and 72.7 ± 2.2%ID/g on days 1, 2, 4, and 7, respectively). Renal uptake peaked at 11.8 ± 0.7%ID/g on day 1 and decreased slowly thereafter. The liver uptake ranged from 5.6 to 6.2%ID/g.

For ^177^Lu-DOTA-IgG, bone marrow uptake remained lower than that of ^177^Lu-DOTA-ATPS mAb and ^177^LuCl_3_ throughout the study (all < 10%ID/g). Uptake of ^177^Lu-DOTA-IgG was similar among the liver, spleen, and kidneys. Renal uptake reached a peak on day 1, while hepatic and splenic uptake increased over time.

Tumor uptake of ^177^Lu-DOTA-ATPS mAb reached 16.0 ± 1.5%ID/g on day 7, which was significantly higher than that of ^177^LuCl_3_ (7.6 ± 0.5%ID/g, *p* < 0.05) and ^177^Lu-DOTA-IgG (8.9 ± 0.5%ID/g, *p* < 0.05). Similarly, on day 4, ^177^Lu-DOTA-ATPS mAb showed greater tumor uptake (12.4 ± 0.4%ID/g) compared to ^177^LuCl_3_ (4.2 ± 0.5%ID/g, *p* < 0.005) and ^177^Lu-DOTA-IgG (8.8 ± 0.5%ID/g, *p* < 0.00005). There was no significant difference in tumor uptake between ^177^LuCl_3_ and ^177^Lu-DOTA-IgG on day 7. The biodistribution patterns of ^177^Lu-DOTA-ATPS mAb, ^177^LuCl_3_, and ^177^Lu-DOTA-IgG in the liver, spleen, kidney, and bone marrow of tumor-bearing mice mirrored those observed in wild-type mice.

In an inhibition study, a high dose of unlabeled ATPS mAb significantly reduced the tumoral uptake of ^177^Lu-DOTA-ATPS mAb from 6.08 ± 1.0%ID/g on day 1 to 3.8 ± 1.1%ID/g (*p* < 0.05).

### 3.5. Radioimmunotherapy, Immunohistochemical Staining, and ^18^F-FDG-PET Imaging in MKN-45 Tumor-Bearing Mice

**Figure 7:**
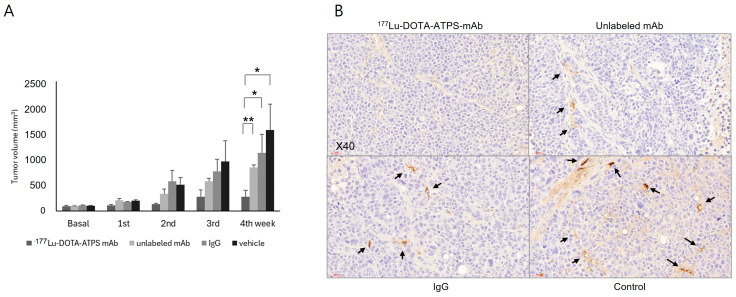

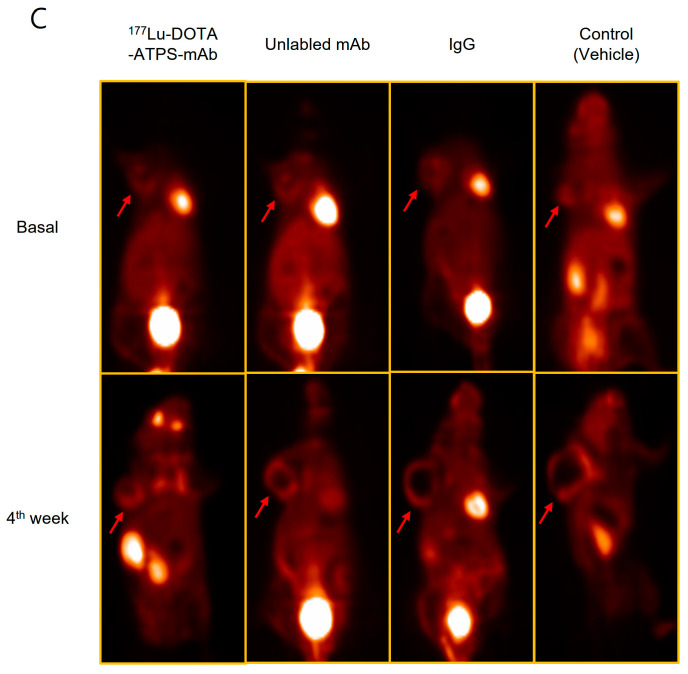
Radioimmunotherapy with ^177^Lu-DOTA-ATPS mAb. (**A**) Tumor growth curve during the 4-week treatment with ^177^Lu-DOTA-ATPS mAb, unlabeled ATPS mAb, IgG, and vehicle. (**B**) Immunohistochemical staining with anti-CD31 antibody for MKN-45 tumors after 4 weeks of treatment. (**C**) ^18^F-FDG PET imaging in mice bearing MKN-45 tumors at baseline and at 4th week of treatment. DOTA, tetraazacyclododecane-1,4,7,10-tetraacetic acid; ATPS, adenosine triphosphate synthase; mAb, monoclonal antibody. *p* < 0.05 *, *p* < 0.01 **, arrows indicate positive staining.

All animals survived until the end of the experiment regardless of treatment (single agent or combination). The therapeutic efficacy of single agents is shown in Figure 7A. No significant difference in initial tumor volume was observed among the groups (^177^Lu-DOTA-ATPS mAb, 92.0 ± 15.5 mm^3^; unlabeled ATPS mAb, 93.6 ± 3.5 mm^3^; IgG, 104.6 ± 6.2 mm^3^; vehicle, 97.1 ± 8.6 mm^3^, *p* > 0.05). After four weeks, tumors treated with ^177^Lu-DOTA-ATPS mAb (269.1 ± 130.4 mm^3^) were significantly smaller than those treated with unlabeled ATPS mAb (836.4 ± 53.1 mm^3^, *p* < 0.01), IgG (1117.1 ± 364.5 mm^3^, *p* < 0.05), or vehicle (1561.4 ± 420.4 mm^3^, *p* < 0.05). While tumors treated with unlabeled ATPS mAb or IgG displayed smaller volumes compared to controls, these differences were not statistically significant. %TGI after four weeks of treatment was 82.8% for ^177^Lu-DOTA-ATPS mAb, 46.4% for unlabeled ATPS mAb, and 28.5% for IgG treatment. Tumor volume in the ^177^Lu-DOTA-ATPS mAb treated group did not significantly change from baseline to the 4th week (*p* > 0.05)

Immunohistochemistry using an anti-CD31 antibody revealed moderate to strong staining in the small vessels of the tumors treated with unlabeled ATPS mAb, IgG, or vehicle (Figure 7B). Conversely, tumors treated with ^177^Lu-DOTA-ATPS mAb exhibited minimal staining.

Representative ^18^F-FDG PET images of mice after four weeks of treatment are shown in Figure 7C. Tumor volume increased in mice treated with unlabeled ATPS mAb, IgG, or vehicle at the 4th week. These tumors also displayed central metabolic defects, indicative of necrotic change. In contrast, tumor volume remained stable in mice treated with ^177^Lu-DOTA-ATPS mAb.

**Figure 8:**
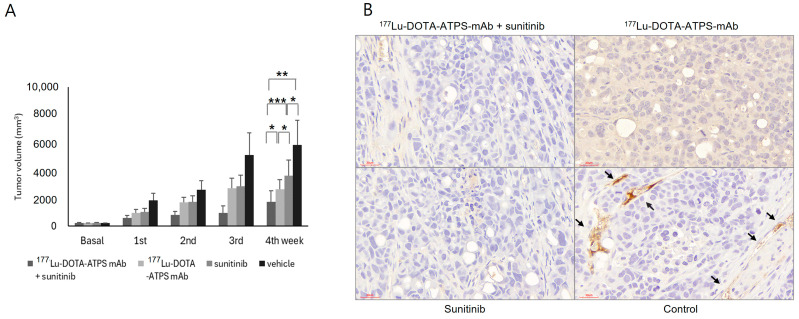

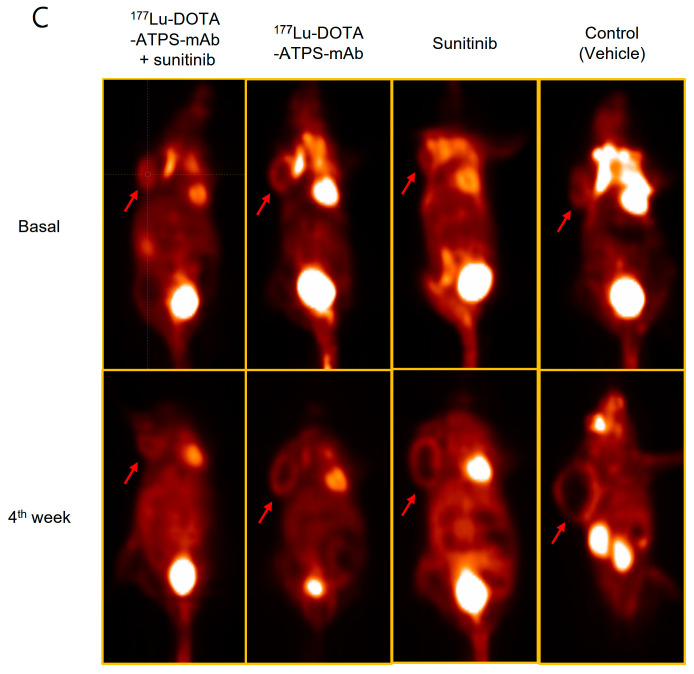
Combination chemo-radioimmunotherapy with sunitinib and ^177^Lu-DOTA-ATPS mAb. (**A**) Tumor growth curve during the 4-week treatment with ^177^Lu-DOTA-ATPS mAb, sunitinib, combination, and vehicle. (**B**) Immunohistochemical staining with anti-CD31 antibody for MKN-45 tumors after 4 weeks of treatment. (**C**) ^18^F-FDG PET imaging in mice bearing MKN-45 tumors at baseline and 4th week of treatment. DOTA, tetraazacyclododecane-1,4,7,10-tetraacetic acid; ATPS, adenosine triphosphate synthase; mAb, monoclonal antibody. *p* < 0.05 *, *p* < 0.01 **, *p* < 0.005 ***, arrows indicate positive staining.

The efficacy of combination therapy is shown in Figure 8A. Initial tumor volume did not differ significantly among groups (^177^Lu-DOTA-ATPS mAb + sunitinib, 209.3 ± 31.3 mm^3^; ^177^Lu-DOTA-ATPS mAb, 210.3 ± 4.2 mm^3^; sunitinib, 207.6 ± 22.9 mm^3^; vehicle, 203.7 ± 18.9 mm^3^, *p* > 0.05). After four weeks, tumors treated with ^177^Lu-DOTA-ATPS mAb (2644.4 ± 703.7 mm^3^, *p* < 0.05), sunitinib (3619.3 ± 1114.0 mm^3^, *p* < 0.05), or the combination (1727.6 ± 793.5 mm^3^, *p* < 0.01) were significantly smaller than those in the vehicle group. Combination therapy with ^177^Lu-DOTA-ATPS mAb and sunitinib demonstrated a greater therapeutic effect compared to either single agent (^177^Lu-DOTA-ATPS mAb, *p* < 0.05; sunitinib, *p* < 0.005). %TGI after four weeks of treatment was 70.3% for the combination, 54.6% for ^177^Lu-DOTA-ATPS mAb alone, and 37.8% for sunitinib alone.

Immunohistochemistry using an anti-CD31 antibody revealed strong staining in the small vessels of tumors from the vehicle group only (Figure 8B). In contrast, tumors treated with ^177^Lu-DOTA-ATPS mAb, sunitinib or the combination exhibited minimal staining. These findings indicate an anti-angiogenic effect of the therapeutic approaches.

Representative ^18^F-FDG PET images of mice after four weeks of treatment are shown in Figure 8C. Tumor volume increased in all groups at the 4th week. Tumors treated with single agents or vehicle were larger than those treated with the combination and displayed central metabolic defects.

## 4. Discussion

In the present study, we aimed to develop a radioimmunotherapy agent targeting tumor angiogenesis. We achieved this by coupling anti-ATPS mAb to ^177^Lu using DOTA as a chelator. Based on cellular uptake results, MKN-45 gastric cancer cells were selected for further development and the evaluation of the therapeutic efficacy of ^177^Lu-DOTA-ATPS mAb. The uptake of ^177^Lu-DOTA-ATPS mAb was specific and inhibited by unlabeled ATPS mAb in both in vitro and in vivo experiments. ^177^Lu-DOTA-ATPS mAb demonstrated a superior therapeutic effect compared to unlabeled ATPS mAb against MKN-45 cells, both in vitro and in vivo. Furthermore, the combination of ^177^Lu-DOTA-ATPS mAb with sunitinib significantly enhanced the therapeutic effect in mice bearing MKN-45 tumors. This enhanced efficacy was also evident in ^18^F-FDG PET imaging and immunohistochemistry analysis. These results suggest that radioimmunotherapy using ^177^Lu-DOTA-ATPS mAb has potential for application in cancer therapy targeting tumor angiogenesis.

^177^Lu is a long-lived (half-life of 6.7 days), β-ray emitting (Emax = 0.49 MeV, range = 670 µm in soft tissue) radioisotope suitable for therapy. The success of ^177^Lu-based radioligand therapy and peptide receptor radionuclide therapy has contributed to the growing popularity of ^177^Lu as a radioisotope for radioimmunotherapy [20]. ^177^Lu-PSMA has shown promising results as a treatment option for metastatic castration-resistant prostate cancer following chemotherapy and hormonal therapy [21]. ^177^Lu-DOTA-TATE effectively reduces tumor growth and stabilizes disease in patients with gastroenteropancreatic well-differentiated neuroendocrine tumors, leading to its establishment as a second- or third-line treatment option [22]. ^177^Lu-labeled trastuzumab, an antibody that targets the HER2 receptor protein, exemplifies radioimmunotherapy using ^177^Lu. This approach demonstrates specific uptake in HER2-positive primary breast cancers and their metastatic sites [23]. Radioimmunotherapy with ^177^Lu has also been explored in anti-angiogenesis cancer treatment. ^177^Lu-labeled TRC105, an antibody targeting CD105, demonstrated tumor uptake in mice with breast cancer. The uptake was 14.3 ± 2.3%ID/g on day 1 and 11.6 ± 6.1%ID/g on day 7, similar to our findings. This approach also inhibited tumor growth and improved survival [24]. These results provide strong support for the use of ^177^Lu-labeled radiopharmaceuticals in radioimmunotherapy.

Among the ^177^Lu-labeled radiopharmaceuticals previously mentioned, ^177^Lu-DOTA-TATE (Lutathera^®^) was approved by the FDA in 2018 for the treatment of somatostatin receptor-positive gastroenteropancreatic neuroendocrine tumors [25]. Additionally, ^177^Lu -PSMA-617 (Pluvicto^®^) was approved in 2022 for the treatment of adult patients with prostate-specific membrane antigen-positive metastatic castration-resistant prostate cancer who have previously been treated with androgen receptor pathway inhibition and taxane-based chemotherapy [26]. Due to the success of ^177^Lu-based therapy, it has recently garnered significant attention. In the near future, we can expect the development of more radiopharmaceutical therapies, not limited to those labeled with ^177^Lu. This progress will provide clinicians with a broader range of treatment options for their cancer patients.

^177^Lu, a radiometal isotope, requires a chelating agent to form a stable complex with antibodies. DOTA, diethylenetriamine pentaacetate (DTPA), and ethylene-diamine-tetraacetic acid (EDTA) are the most common chelators for radiometal isotopes [27,28]. In our preliminary study, ^177^Lu-DOTA-ATPS mAb exhibited excellent labeling efficiency (around 99.0%) and stability in repeated experiments. Conversely, the labeling efficiency of ^177^Lu-DTPA-ATPS mAb was significantly lower. This aligns with previous reports [29,30]. We opted for DOTA-based mAb on these findings.

DOTA, a macrocyclic chelator offers greater in vivo stability compared to acyclic chelators like DTPA and EDTA (“macrocyclic effect”) [27]. This translates to its recommendation for labeling ^177^Lu. As shown in this study, free ^177^Lu accumulates significantly in bone marrow (72.7% for wild-type mice and 69.5% for tumor-bearing mice on day 7). In vivo dissociation of ^177^Lu-DOTA-ATPS mAb can decrease therapeutic efficacy and increases bone marrow toxicity. Despite maintaining high in vitro stability (85.5% at 37 °C on day 7) in serum, ^177^Lu-DOTA-ATPS mAb exhibited significant bone marrow uptake of free ^177^Lu (27.0% for wild-type mice and 39.3% for tumor-bearing mice on day 7). This can be explained by two factors: first, the presence of various blood proteins like transferrin and albumin that strongly bind to ^177^Lu and, furthermore, the dilution of ^177^Lu-DOTA-ATPS mAb in vivo [28]. Overcoming this limitation is crucial, as researchers are actively developing new chelators [31]. Further studies to improve the in vivo stability of ^177^Lu-DOTA-ATPS mAb are required.

ATPS is normally located in the inner mitochondrial membranes as part of the mitochondrial respiratory complex. It participates in ATP production using a proton gradient generated by mitochondrial respiratory complex I-IV [32]. Interestingly, ATPS can also be found on the surface of some cancer and endothelial cells, known as ectopic ATPS. This ectopic ATPS can be a binding site for angiostatin [3]. Therefore, ATPS could serve as a novel target for anti-angiogenic cancer therapies. As previously demonstrated, the anti-ATPS mAb used in this study can target tumor vasculature and cancer cells [9]. In this study, ^177^Lu-DOTA-ATPS mAb showed a significant inhibitory effect on MKN-45 gastric cancers. TGI of ^177^Lu-DOTA-ATPS mAb (82.8%) was greater than that of unlabeled ATPS mAb (46.6%). Additionally, immunohistochemistry with an anti-CD31 antibody revealed minimal staining in tumors treated with ^177^Lu-DOTA-ATPS mAb (Figure 7B). Similarly, minimal anti-CD31 staining was observed in tumors treated with either single-agent sunitinib or combination therapy (Figure 8B). These findings indicate that the anticancer efficacy of these therapeutic strategies is likely mediated through targeting tumor angiogenesis.

Combination therapy offers a significant advantage by enhancing anticancer effects while potentially reducing side effects compared to single-agent treatment. In this study, combination therapy demonstrated a synergistic increase in therapeutic efficacy (TGI = 70.3%) compared to sunitinib alone (37.8%). Furthermore, ^177^Lu-DOTA-ATPS mAb alone (TGI = 54.6%) also demonstrated a greater therapeutic effect than sunitinib alone. This finding suggests promising potential for the clinical application of ^177^Lu-DOTA-ATPS mAb in the future. Chemotherapeutic agents, such as tyrosine kinase inhibitors, have been known to show a broad spectrum of adverse effects in both the hematologic system and nonhematologic systems [33]. ^177^Lu-based radiopharmaceutical therapies have also been associated with various side effects, some of which are serious hematologic diseases [34,35]. Such side effects could obstruct the appropriate treatment of cancer patients, leading to a reduction in dosage or discontinuation of therapeutic agents. Based on the results of this study, combining ^177^Lu-radioimmunotherapy with conventional chemotherapy could decrease the therapeutic doses of each treatment, leading to fewer adverse effects than single-agent therapy. However, further clinical studies should be conducted.

^177^Lu decays by emitting two γ-rays (208 and 113 KeV), which are ideal for gamma camera imaging. While we employed ^18^F-FDG PET for tumor visualization in this study, whole-body gamma camera imaging could directly visualize or predict the biodistribution of ^177^Lu-DOTA-ATPS mAb. A limitation of this study is the lack of a small animal gamma camera, which prevented us from performing this complementary imaging modality.

We were unable to achieve tumor-free survival as tumors persistently grew in all groups, whether they were subjected to single or combination treatments. In the combination treatment group, tumors from two mice did not exhibit growth until the fourth week of therapy, although they did not completely disappear. In contrast, all tumors in the other groups showed significant growth by the fourth week of treatment. In this study, we initiated treatments when the tumors reached approximately 200 mm^3^ in size. For a more accurate evaluation of tumor-free survival, treatments should ideally be started earlier. This is another limitation of the study.

In our previous study [9], we categorized six types of cancer cells into two groups based on their membranous ATPS expression, as determined by Western blot analysis and immunofluorescence microscope findings. From these, we chose MDA-MB-231 (which has high ATPS expression) and PC-3 (which has low ATPS expression) for further comparison. We evaluated these cells using in vitro cellular uptake, binding, and in vivo tumor uptake with ^89^Zr-labeled ATPS mAb and positron emission tomography imaging (utilizing the same mAb as in this study). The MDA-MB-231 cells exhibited significantly higher cellular uptake, binding capability (Kd), and in vivo tumor uptake than the PC-3 cells. Based on these results, we hypothesized that cells demonstrating higher in vitro cellular uptake would inevitably show higher in vivo tumor uptake, leading to increased therapeutic efficacy. While it is a well-established concept in clinical radioimmunotherapy to predict therapeutic outcomes using diagnostic radiopharmaceuticals with the same antibodies, it would be beneficial to evaluate the ATPS expression of cancer cells concurrently with cellular uptake. As an alternative, comparing the therapeutic effects between tumors with high ATPS expression and those with lower ATPS expression could aid in drawing more credible conclusions. This is also acknowledged as a limitation of this study.

Despite encouraging preclinical results, this study represents early-stage research. Further technical refinements are necessary before clinical trials with ^177^Lu-DOTA-ATPS mAb can be initiated.

## Data Availability

The data on this study are available in the manuscript. Further inquiries can be directed to the corresponding author.
